# Reconsideration of Information-Theoretic Principles—Perspective from the Dual Probability Distribution

**DOI:** 10.3390/e28060681

**Published:** 2026-06-12

**Authors:** Yoshikazu Ohtaki, Tomomi Nakamura, Hiroshi-H. Hasegawa, Tatsuaki Wada

**Affiliations:** 1 Department of Mathematics and Informatics, Ibaraki University, Mito 310-8512, Japan; yoshi_oojp@yahoo.co.jp (Y.O.); tomomi.nakamura.aiw@gmail.com (T.N.); 2 Region of Electrical and Electronic Systems Engineering, Ibaraki University, Hitachi 316-8511, Japan; tatsuaki.wada.to@vc.ibaraki.ac.jp

**Keywords:** divergence, Pythagorean theorem, Legendre transformation, Massieu potential, maximum entropy principle, minimum Massieu potential principle, Sanov’s Lemma, large deviation principle, Robbins’ bound, information geometry

## Abstract

We reconsider information-theoretic principles, such as the maximum entropy principle/minimum Massieu potential principle, from the perspective of the dual probability distribution. This is introduced through Sanov’s Lemma for the multinomial distribution. The dual correspondence becomes asymptotically manifest. The Massieu potential is rewritten as the Kullback–Leibler divergence between the dual probability distribution and the dual reference distribution. Similarly, the dual potential is rewritten as the cumulant generating function with respect to the dual reference distribution. This perspective gives us new insight into information-theoretic principles. As the dual probability distribution naturally arises in data sampling, we anticipate that this new perspective will play a significant role in data analysis.

## 1. Introduction

In information geometry, probability distributions are treated as points in a parameter space, and the ‘distance’ between them is treated as divergence [1,2]. The well-known Kullback–Leibler (KL) divergence/relative entropy is not symmetric with respect to the exchange of two probability distributions, meaning it does not satisfy the axiom of distance.

Symmetry plays a significant role in physics. Attempts to introduce symmetric divergences, such as the Jensen–Shannon divergence [3], are significant. However, Amari et al. focused on the hidden dual structure behind asymmetric divergences instead, revealing the dual flat structure in the Pythagorean theorem [1,2].

The dual probability distributions, also known as ‘statistically dual distributions’ [4], refer to a pair of distributions in which the roles of the parameter and the stochastic variable are interchanged. For example, in a canonical distribution $\rho \left(E\left(x\right)|\beta \right)=\exp\left[-\beta E\left(x\right)\right]$ with a constant temperature T, the parameter is the inverse temperature $\beta =\frac{1}{kT}$, and the stochastic variable is the energy $E\left(x\right)$ of a state x. We can consider its dual distribution $\rho \left(\beta \left(y\right)|E\right)$, where these roles are swapped: the energy E is treated as the parameter and the inverse temperature β(y) of a state as the stochastic variable y. At the same time, the temperature in a canonical distribution is usually a fixed number that does not fluctuate. In contrast, in a real system, in the case of a finite heat bath, its temperature can fluctuate. In this case, the parameter is given as the average of the fluctuating temperature. The dual probability distribution accounts for the variability of the parameter that is fixed in the thermodynamic limit.

In Section 2, we provide a brief review of the information geometry of the exponential family on the n-dimensional simplex $S_{n}$, with a given reference distribution. The Massieu potential [5] becomes the cumulant generating function with respect to the reference distribution. The dual parameter vector is generated as an average of the Massieu potential via Legendre transformation, and the dual potential becomes the KL divergence between the probability distribution and the reference distribution.

In Section 3, we introduce the concept of a dual probability distribution. According to Sanov’s Lemma [6], the dual potential can be interpreted as an exponent of the sampling distribution generated by the reference distribution. The integral representation of this exponent introduces new stochastic variables that follow a dual probability distribution. This yields a dual-symmetric correspondence asymptotically. The dual potential can be rewritten as a cumulant generating function with respect to the dual reference distribution. The parameter vector can be generated as an average from the dual potential using the inverse Legendre transformation. The Massieu potential can be expressed as the KL divergence between the dual probability distribution and the dual reference distribution.

Our derivation is based on Stirling’s approximation. Therefore, the Massieu/dual potential requires modification for finite N. Upon estimating the correction term, we found that the O(logN/N) term arising from Stirling’s formula is negligible, and that the correction to the Massieu/dual potential is a convex function of order O(1/N).

In Section 4, we reconsider the maximum entropy principle and its dual in the context of the dual probability distribution. The maximum entropy principle is derived asymptotically from the Pythagorean theorem based on the orthogonality between the canonical geodesic and the iso-energy surface. For the dual principle, the minimum Massieu potential principle, we introduce an explicit dual canonical distribution. The exponent of this distribution is expressed by one dual parameter and a dual energy function. This principle is also derived asymptotically from the Pythagorean theorem based on the orthogonality between the dual canonical geodesic and the dual-iso-energy surface.

We expect new insights to play an important role in data analysis, given that dual probability distributions naturally arise in data sampling. Specifically, we intend to apply our argument to the analysis of time series for simple chaotic systems [7], the probability distribution of which evolves according to a gradient flow equation [8,9,10]. These decay modes are known as the Ruelle–Pollicott resonances. Although originally introduced in connection with decay in classical chaotic systems, it has recently attracted attention in relation to irreversibility in quantum many-body chaos [11].

In Section 5, we present the results of applying our discussion to a simple chaotic map system. We simulate the time evolution for several initial states. In particular, we focus on the time evolution of the Massieu potential to clarify its information-geometric meaning.

In Section 6, we summarize these duality correspondences and comment.

## 2. Exponential Family in n-Dimensional Simplex

### 2.1. Exponential Family in n-Dimensional Simplex

The exponential family is a class of probability distributions commonly encountered in information geometry. For simplicity, we will mainly deal with $S_{n}$ in this section. In data analysis, a probability distribution is represented as a histogram of empirical data. In practice, any probability distribution can be approximated by $S_{n}$.

A probability distribution in $S_{n}$ is written as the following representation of the exponential family in information geometry [1,2],

(1) $$p_{x}\left(\theta \right)=exp\left[\theta \cdot \delta_{x}-\psi \left(\theta \right)\right]{\bar{p}}_{x},$$

where a stochastic variable, x=0,1,…,n, n-dimensional parameter vector: $\theta =\left(\theta_{1},\;\theta_{2},\ldots ,\theta_{n}\right)$ and $\theta \cdot \delta_{x}\equiv \sum_{i=1}^{n}\theta_{i}\delta_{x,i}$. And ${\bar{p}}_{x}$ is a reference distribution, ${\bar{p}}_{x}>0\;and\;\sum_{x}{\bar{p}}_{x}=1.$

To satisfy the normalization condition, $\sum_{x}p_{x}\left(\theta \right)=1,$

(2) $$exp\left[\psi \left(\theta \right)\right]=E_{\bar{p}}\left[exp\left[\theta \cdot \delta_{x}\right]\right],$$

where we write the expectation value with respect to distribution $p_{x}$ as, $E_{p_{x}}\left[\cdot \right]\equiv \sum_{x=0}^{n}\cdot p_{x}.$ Then, the Massieu potential is written as the cumulant generating function with respect to the reference distribution,

(3) $$\psi \left(\theta \right)=\log E_{\bar{p}}\left[exp\left[\theta \cdot \delta_{x}\right]\right]=-\log\left(\frac{p_{0}}{{\bar{p}}_{0}}\right).$$

The i-th element of the n-dimensional parameter vector θ is written as,

(4) $$\theta_{i}=\log\left(\frac{p_{i}}{{\bar{p}}_{i}}\frac{{\bar{p}}_{0}}{p_{0}}\right).$$

Since the Massieu potential is a convex function, the Hessian,

(5) $$\partial_{\theta_{i}}\partial_{\theta_{j}}\psi \left(\theta \right),$$

is the positive definite matrix.

### 2.2. Legendre Transformation

The dual parameters are defined by the Legendre transformation based on the Massieu potential,

(6) $$\theta_{i}^{*}=\partial_{\theta_{i}}\psi \left(\theta \right).$$

They are written as the average of $\delta_{x,i}$ with respect to $p_{x}$,

(7) $$\theta_{i}^{*}=E_{p_{x}}\left[\delta_{x,i}\right]=p_{i}.$$

The dual potential is also defined as,

(8) $$\varphi \left(\theta^{*}\right)=\theta \cdot \theta^{*}-\psi \left(\theta \right)=D_{KL}\left[p:\bar{p}\right],$$

where the KL divergence with respect to $p_{x}$,

(9) $$D_{KL}\left[p:\bar{p}\right]=\sum_{x=0}^{n}p_{x}\log\left(\frac{p_{x}}{{\bar{p}}_{x}}\right),$$

which reduces to the well-known negentropy for the uniform reference distribution up to a constant.

### 2.3. Divergences

In the information geometry, a probability distribution p(θ) in $S_{n}$ is represented as a point θ in the n dimensional parameter space. The information distance between two probability distributions, $p(\theta_{A})$ and $p(\theta_{B})$, is given by the Bregman divergence based on the convex function, the Massieu potential $\psi \left(\theta \right)$,

(10) $$D_{\psi }\left[\theta_{A}:\theta_{B}\right]=\psi \left(\theta_{A}\right)-\psi \left(\theta_{B}\right)-\left(\theta_{A}-\theta_{B}\right)\cdot \nabla_{\theta_{B}}\psi \left(\theta_{B}\right).$$

Using the dual potential, the Bregman divergence is rewritten as follows,

(11) $$D\left[\theta_{A}:\theta_{B}^{*}\right]=\psi \left(\theta_{A}\right)+\varphi \left(\theta_{B}^{*}\right)-\theta_{A}\cdot \theta_{B}^{*}.$$

We mainly use this divergence without any subscript in this article, since it has a dual symmetric form under the Legendre transformation.

The divergence is related to the KL divergence as,

(12) $$D\left[\theta_{A}:\theta_{B}^{*}\right]=D_{KL}\left[p\left(\theta_{B}\right):p\left(\theta_{A}\right)\right],$$

where we used the following cross-entropy with the reference distribution,

(13) $$-E_{p\left(\theta_{B}\right)}\left[\log\left(\frac{p\left(\theta_{A}\right)}{\bar{p}}\right)\right]=-\theta_{A}\cdot \theta_{B}^{*}+\psi \left(\theta_{A}\right).$$

## 3. Dual Probability Distribution

### 3.1. Dual Probability Distribution

We will consider how the parameter vector, θ, fluctuates. Although parameters are usually treated as constants, we will demonstrate that they can be interpreted as the expected value of certain stochastic variables.

The dual potential, the KL divergence with respect to $p_{x}$, is considered as the logarithm of the multinomial distribution in N independent trials,

(14) $$-\frac{1}{N}\log P_{MD}\left(Np|\bar{p}\right)=-\frac{1}{N}\log\left[\frac{N!}{N_{0}!N_{1}!\ldots N_{n}!}{\bar{p}}_{0}^{N_{0}}{\bar{p}}_{1}^{N_{1}}\ldots {\bar{p}}_{n}^{N_{n}}\right]=D_{KL}\left[p:\bar{p}\right]+O\left(\frac{\log N}{N}\right),$$

where $p_{x}=N_{x}/N$. The derivation is based on the Stirling formula for large N [2]. This relation is known as Sanov’s Lemma [6] in the large deviation theory [12].

Using the integral form of the Gamma function,

(15) $$N!=\Gamma \left(N+1\right)=\int_{-\infty }^{\infty }e^{Ny}e^{y-e^{y}}dy,$$

we can introduce dual stochastic variables, $y_{x}\;\left(x=0,\;1,\ldots ,n\right)$.

We can rewrite the dual potential as the cumulant generating function with respect to the dual stochastic variables up to a constant and a negligible function,

(16) $$\varphi \left(\theta^{*}\right)-{∆\varphi }_{N}\left(\theta^{*}\right)=\frac{1}{N}\log E_{\bar{P}\left(y\right)}\left[e^{N\theta^{*}\cdot \tilde{y}}\right]+c_{N},$$

where ${\tilde{y}}_{i}=y_{i}-y_{0}\;\left(i=1,\ldots ,n\right)$, the dual reference distribution,

(17) $$\bar{P}\left(y\right)=\frac{N^{N}}{N!}{\bar{p}}_{0}^{N}e^{{Ny}_{0}}e^{\sum_{x}\{y_{x}+\log(N{\bar{p}}_{x})-e^{y_{x}+\log(N{\bar{p}}_{x})}\}},$$

and the constant $c_{N}$ and a convex function ${∆\varphi }_{N}(\theta^{*})$ defined as follows,

(18) $$c_{N}\equiv -\log{\bar{p}}_{0}-\frac{n+1}{2N}\log\left(2\pi N\right)+1-\log N+\frac{1}{N}\log\left(N!\right),$$

(19) $${∆\varphi }_{N}\left(\theta^{*}\right)\equiv -\frac{1}{N}\sum_{x=0}^{n}\left[\log\left({(Np}_{x})!\right)-\left\{{Np}_{x}\log\left({Np}_{x}\right)-{Np}_{x}\right\}-\frac{1}{2}\log\left(2\pi N\right)\right]\;=-\frac{1}{N}\sum_{x=0}^{n}\left\{\frac{1}{2}\log p_{x}+\lambda_{N_{x}}\right\}=O\left(\frac{1}{N}\right).$$

where $\frac{1}{12N+1}<\lambda_{N}<\frac{1}{12N}$ is known as Robbins’ bound [13]. We note that ${∆\varphi }_{N}\left(\theta^{*}\right)$ is a convex function for N large so that $\varphi \left(\theta^{*}\right)-{∆\varphi }_{N}\left(\theta^{*}\right)$ is interpreted as a modified dual potential. These derivations are shown in Appendix A.

Since the modified dual potential is the cumulant generating function up to a constant, the inverse Legendre transformation gives the following modified parameter vector as averages of the dual stochastic variables,

(20) $$\theta -∆\theta =\nabla_{\theta^{*}}\left(\varphi \left(\theta^{*}\right)-{∆\varphi }_{N}\left(\theta^{*}\right)\right)=E_{P\left(y|\theta^{*}\right)}\left[\tilde{y}\right],$$

where the dual probability distribution,

(21) $$P\left(y|\theta^{*}\right)=e^{N\{\theta^{*}\cdot \tilde{y}-\varphi \left(\theta^{*}\right)+{∆\varphi }_{N}\left(\theta^{*}\right)+c_{N}\}}\bar{P}\left(y\right).$$

The modified Massieu potential is rewritten as the following scaled KL divergence between $P\left(y|\theta^{*}\right)\;and\;\bar{P}\left(y\right)$ up to a constant,

(22) $$\psi \left(\theta \right)-∆\psi_{N}\left(\theta \right)=E_{P\left(y|\theta^{*}\right)}\left[\theta^{*}\cdot \tilde{y}-\varphi \left(\theta^{*}\right)+{∆\varphi }_{N}\left(\theta^{*}\right)+c_{N}\right]-c_{N}\;=\frac{1}{N}D_{KL}\left[P\left(y|\theta^{*}\right):\bar{P}\left(y\right)\right]-c_{N}.$$

The Massieu potential can be considered as the information/negentropy of the dual stochastic variables.

The dual stochastic variables represent fluctuations in the distribution of the data sample. The order of the deviation is $O(1/\sqrt{N})$. From the Central Limit Theorem, the distribution becomes a normal distribution:

(23) $$P\left(y\right)≅N\left(\bar{y},\Sigma \right),$$

where ${\bar{y}}_{x}=\log p_{x}-\log{\bar{p}}_{x}+1$, and $\Sigma_{x,x’}=\delta_{x,x^{\prime }}/(Np_{x}).$

Although fluctuation is negligible for large N, it is important for finite N. For example, the inverse of temperature is the typical parameter in the canonical distribution. Its fluctuation is negligible for a large reservoir in the thermodynamical limit. But the temperature can fluctuate in a finite system.

### 3.2. Divergence

The KL divergence between $P\left(y|\theta_{A}^{*}\right)$ and $P\left(y|\theta_{B}^{*}\right)$ is rewritten by the modified potentials as,

(24) $$\frac{1}{N}D_{KL}\left[P\left(y|\theta_{A}^{*}\right):P\left(y|\theta_{B}^{*}\right)\right]=\psi \left(\theta_{A}\right)-∆\psi_{N}\left(\theta_{A}\right)+\varphi \left(\theta_{B}^{*}\right)-{∆\varphi }_{N}\left(\theta_{B}^{*}\right)-\left(\theta_{A}-∆\theta_{A}\right)\cdot \theta_{B}^{*},$$

where we used (22) for the modified Massieu potential and the following dual cross-entropy derived from (20) and (21),

(25) $$-\frac{1}{N}E_{P\left(y|\theta_{A}^{*}\right)}\left[\log\frac{P\left(y|\theta_{B}^{*}\right)}{\bar{P}\left(y\right)}\right]=-(\theta_{A}-∆\theta_{A})\cdot \theta_{B}^{*}+\varphi \left(\theta_{B}^{*}\right)-{∆\varphi }_{N}\left(\theta_{B}^{*}\right)-c_{N}.$$

The KL divergence between the dual probability distributions, $P\left(y|\theta_{A}^{*}\right)$ and $P\left(y|\theta_{B}^{*}\right)$, is given as the following modified divergence between $\theta_{B}$ and $\theta_{A}^{*}$ as,

(26) $$\frac{1}{N}D_{KL}\left[P\left(y|\theta_{A}^{*}\right):P\left(y|\theta_{B}^{*}\right)\right]=D\left[\theta_{A}:\theta_{B}^{*}\right]-{∆D}_{N}\left[\theta_{A}:\theta_{B}^{*}\right],$$

where ${∆D}_{N}\left[\theta_{A}:\theta_{B}^{*}\right]$ is a Bregman divergence while ${∆\varphi }_{N}\left(\theta^{*}\right)$ is a convex function with respect to $\theta^{*}$,

(27) $${∆D}_{N}\left[\theta_{A}:\theta_{B}^{*}\right]\equiv ∆\psi_{N}\left(\theta_{A}\right)+{∆\varphi }_{N}\left(\theta_{B}^{*}\right)-∆\theta_{A}\cdot \theta_{B}^{*}=O\left(\frac{1}{N}\right).$$

The KL divergence between $P\left(y|\theta_{A}^{*}\right)$ and $P\left(y|\theta_{B}^{*}\right)$ asymptotically becomes the dual KL divergence between $p\left(\theta_{B}\right)$ and $p\left(\theta_{A}\right)$ in the limit of large N,

(28) $$\underset{N\to \infty }{\lim}D_{KL}\left[P\left(y|\theta_{A}^{*}\right):P\left(y|\theta_{B}^{*}\right)\right]=D_{KL}\left[p\left(\theta_{B}\right):p\left(\theta_{A}\right)\right].$$

### 3.3. Pythagorean Theorem

The Pythagorean theorem for the KL divergences between the dual probability distributions is given as follows,

(29) $$\begin{array}{c} \frac{1}{N}D_{KL}[P\left(y|\theta_{A}^{*}\right):P\left(y|\theta_{C}^{*}\right)]-\frac{1}{N}D_{KL}[P\left(y|\theta_{A}^{*}\right):P\left(y|\theta_{B}^{*}\right)]-\frac{1}{N}D_{KL}[P\left(y|\theta_{B}^{*}\right):P\left(y|\theta_{C}^{*}\right)]\\ =D\left[\theta_{A}:\theta_{c}^{*}\right]-{∆D}_{N}\left[\theta_{A}:\theta_{c}^{*}\right]-D\left[\theta_{A}:\theta_{B}^{*}\right]+{∆D}_{N}\left[\theta_{A}:\theta_{B}^{*}\right]-D\left[\theta_{B}:\theta_{C}^{*}\right]+{∆D}_{N}\left[\theta_{B}:\theta_{C}^{*}\right]\\ =\left(\theta_{A}-∆\theta_{A}-\theta_{B}+∆\theta_{B}\right)\cdot \left(\theta_{B}^{*}-\theta_{C}^{*}\right)=0, \end{array}$$

where the parameter geodesic is modified.

## 4. Maximum Entropy Principle and the Dual Principle

### 4.1. Maximum Entropy Principle

We introduce a canonical parameter following the one-parameter geodesic,

(30) $$\theta_{can}\left(\beta \right)-∆\theta_{can}\left(\beta \right)=-\beta E,$$

that is corresponding to the canonical distribution in physics,

(31) $$p\left(\theta_{can}\left(\beta \right)-∆\theta_{can}\left(\beta \right)\right)=exp\left[-\beta E_{x}+F(\beta )\right]{\bar{p}}_{x}.$$

The parameter β is the inverse temperature included in the Boltzmann constant, $E_{x}$ is the n+1-dimensional energy function, $E_{x}=E\cdot \delta_{x}\equiv \sum_{i=1}^{n}E_{i}\delta_{x,i}$ and $E=\left(E_{1},\ldots E_{n}\right)$ where we choose $E_{0}=0$ for which the free potential is given as the negative Massieu potential,

(32) $$F\left(\beta \right)\equiv \beta F\left(\beta \right)=-\psi \left(\theta_{can}\left(\beta \right)-∆\theta_{can}\left(\beta \right)\right).$$

Note that $F\left(\beta \right)$ is the free energy and $F\left(\beta \right)$ is the dimensionless potential.

The orthogonal condition for the Pythagorean theorem in (29) to hold can be rewritten as for $\theta_{A}-∆\theta_{A}=\theta_{can}\left(\alpha \right)-∆\theta_{can}\left(\alpha \right)$, $\theta_{B}-∆\theta_{B}=\theta_{can}\left(\beta \right)-∆\theta_{can}\left(\beta \right),\;\theta_{B}^{*}=\theta_{can}^{*}\left(\beta \right)=p\left(\theta_{can}\left(\beta \right)\right)\;\mathrm{and}\;\theta_{C}^{*}=\theta^{*}=p(\theta )$,

(33) $$\left(\alpha -\beta \right)E\cdot \left(\theta_{can}^{*}\left(\beta \right)-\theta^{*}\right)=\left(\alpha -\beta \right)\left(E\cdot p\left(\theta_{can}\left(\beta \right)\right)-E\cdot p\left(\theta \right)\right)=0.$$

**Figure 1:**
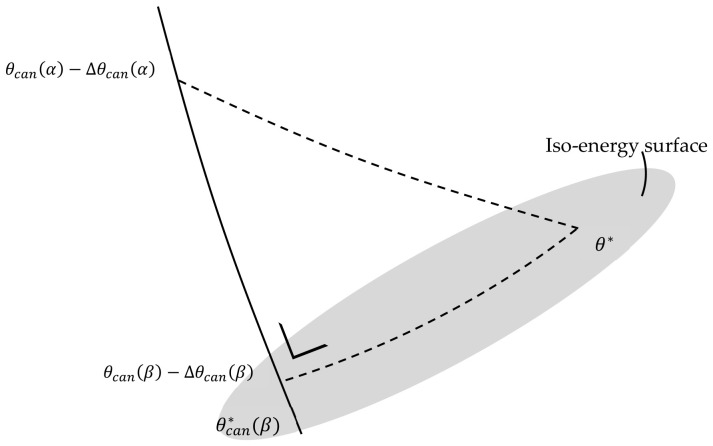
The canonical geodesic, $\theta_{can}\left(\alpha \right)-∆\theta_{can}\left(\alpha \right)-\theta_{can}\left(\beta \right)+∆\theta_{can}\left(\beta \right)\propto E$ is perpendicular to iso-energy surface, $E\cdot \theta_{can}^{*}\left(\beta \right)=E\cdot \theta^{*}$.

This implies that the canonical geodesic is perpendicular to the iso-energy surface, as is shown in Figure 1. Under this orthogonal condition, the Pythagorean theorem holds as,

(34) $$\;\;\;\;\;\;\;\;\;\;\;\;\;\;\;\frac{1}{N}D_{KL}\left[P\left(y|\theta_{can}^{*}\left(\alpha \right)\right):P\left(y|\theta^{*}\right)\right]\;=\frac{1}{N}D_{KL}\left[P\left(y|\theta_{can}^{*}\left(\alpha \right)\right):P\left(y|\theta_{can}^{*}\left(\beta \right)\right)\right]+\frac{1}{N}D_{KL}\left[P\left(y|\theta_{can}^{*}\left(\beta \right)\right):P\left(y|\theta^{*}\right)\right].$$

The maximum entropy principle is derived under the iso-energy condition. From (34), the following inequality is derived,

(35) $$\;\;\;\;\;\;\;\;\;\;\;\;\;\;\;\frac{1}{N}D_{KL}\left[P\left(y|\theta_{can}^{*}\left(\alpha \right)\right):P\left(y|\theta^{*}\right)\right]\geq \frac{1}{N}D_{KL}\left[P\left(y|\theta_{can}^{*}\left(\alpha \right)\right):P\left(y|\theta_{can}^{*}\left(\beta \right)\right)\right].$$

By using (20) and (30),

(36) $$\alpha E\cdot \theta^{*}-\varphi \left(\theta^{*}\right)+{∆\varphi }_{N}\left(\theta^{*}\right)\leq \alpha E\cdot \theta_{can}^{*}\left(\beta \right)-\varphi \left(\theta_{can}^{*}\left(\beta \right)\right)+{∆\varphi }_{N}\left(\theta_{can}^{*}\left(\beta \right)\right).$$

From the iso-energy condition, we rigorously obtained the following inequality,

(37) $$-\varphi \left(\theta^{*}\right)+{∆\varphi }_{N}\left(\theta^{*}\right)\leq -\varphi \left(\theta_{can}^{*}\left(\beta \right)\right)+{∆\varphi }_{N}\left(\theta_{can}^{*}\left(\beta \right)\right).$$

The maximum entropy principle is derived in the limit of large N,

(38) $$-\varphi \left(\theta^{*}\right)\leq -\varphi \left(\theta_{can}^{*}\left(\beta \right)\right).$$

We note that the dual potential is the negative entropy for the uniform reference distribution up to a constant.

### 4.2. Minimum Massieu-Potential Principle

We introduce a dual canonical parameter following the one-parameter dual-geodesic,

(39) $$\vartheta_{can}^{*}\left(\kappa \right)\cdot \tilde{y}=\kappa \vartheta_{can}^{*}\left(\lambda \right)\cdot \tilde{y}/\lambda =-\kappa E\left(\tilde{y}\right),$$

that is corresponding to the dual canonical distribution,

(40) $$P\left(y|\vartheta_{can}^{*}\left(\kappa \right)\right)=exp\left[-\kappa E\left(\tilde{y}\right)-\varphi \left(\vartheta_{can}^{*}\left(\kappa \right)\right)+∆\varphi \left(\vartheta_{can}^{*}\left(\kappa \right)\right)+c_{N}\right]\bar{P}(y).$$

Two dual canonical parameters are connected through a scale transformation in (39), $\vartheta_{can,i}^{*}\left(\kappa \right)=\kappa \vartheta_{can,i}^{*}\left(\lambda \right)/\lambda \;\left(i=1,\ldots ,n\right)$. However, $\vartheta_{can,x}^{*}\left(\kappa \right)=$ $p_{can,x}\left(\vartheta_{can}\left(\kappa \right)\right)$ in $S_{n}$, the normalization of $p_{can,x}\left(\vartheta_{can}\left(\kappa \right)\right)$ is the same as $p_{can,x}\left(\vartheta_{can}\left(\lambda \right)\right)$ due to the following relation between their respective zero-components,

(41) $$\vartheta_{can,0}^{*}\left(\kappa \right)=1-\frac{\kappa }{\lambda }\left(1-\vartheta_{can,0}^{*}\left(\lambda \right)\right)>0.$$

The orthogonal condition in the Pythagorean theorem can be rewritten as for $\theta_{A}-∆\theta_{A}=\theta -∆\theta =E_{P\left(y|\theta^{*}\right)}[\tilde{y}]$, $\theta_{B}-∆\theta_{B}=\vartheta_{can}\left(\lambda \right)-∆\vartheta_{can}\left(\lambda \right)=E_{P\left(y|\vartheta_{can}^{*}\left(\lambda \right)\right)}\left[\tilde{y}\right]\;,$ $\theta_{B}^{*}=\vartheta_{can}^{*}\left(\lambda \right),$ and $\theta_{C}^{*}=\vartheta_{can}^{*}\left(\kappa \right)=\kappa \vartheta_{can}^{*}\left(\lambda \right)/\lambda \;$,

(42) $$\left(\theta -∆\theta -\vartheta_{can}\left(\lambda \right)+∆\vartheta_{can}\left(\lambda \right)\right)\cdot \vartheta_{can}^{*}\left(\lambda \right)\left(1-\kappa /\lambda \right)\;=\left(E_{P\left(y|\vartheta_{can}^{*}\left(\lambda \right)\right)}\left[E\left(\tilde{y}\right)\right]-E_{P\left(y|\theta^{*}\right)}[E\left(\tilde{y}\right)]\right)\left(\lambda -\kappa \right)=0.$$

**Figure 2:**
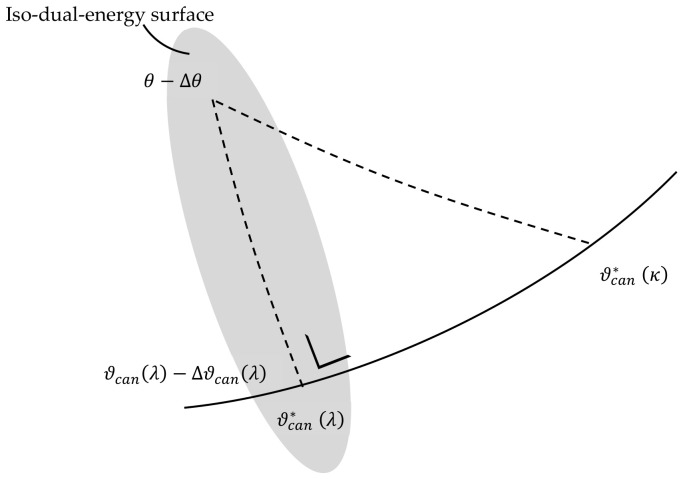
The dual canonical geodesic, $(\vartheta_{can}^{*}\left(\kappa \right)-\vartheta_{can}^{*}\left(\lambda \right))\cdot \tilde{y}\propto E\left(\tilde{y}\right)$ is perpendicular to the iso-dual-energy surface.

This implies that the canonical dual geodesic is perpendicular to the iso-dual-energy surface, as is shown in Figure 2. Under this orthogonal condition, the Pythagorean theorem holds as,

(43) $$\;\;\;\;\;\;\;\;\;\;\;\;\;\;\;\frac{1}{N}D_{KL}\left[P\left(y|\theta^{*}\right):P\left(y|\vartheta_{can}^{*}\left(\kappa \right)\right)\right]\;=\frac{1}{N}D_{KL}\left[P\left(y|\theta^{*}\right):P\left(y|\vartheta_{can}^{*}\left(\lambda \right)\right)\right]+\frac{1}{N}D_{KL}[P\left(y|\vartheta_{can}^{*}\left(\lambda \right)\right):P\left(y|\vartheta_{can}^{*}\left(\kappa \right)\right)].$$

The minimum Massieu-potential principle is derived under the iso-dual-energy condition. From the Pythagorean theorem (43), the following inequality is derived,

(44) $$\;\;\;\;\;\;\;\;\;\;\;\;\;\;\;\;\frac{1}{N}D_{KL}\left[P\left(y|\theta^{*}\right):P\left(y|\vartheta_{can}^{*}\left(\kappa \right)\right)\right]\geq \frac{1}{N}D_{KL}[P\left(y|\vartheta_{can}^{*}\left(\lambda \right)\right):P\left(y|\vartheta_{can}^{*}\left(\kappa \right)\right)].$$

After canceling $\varphi \left(\vartheta_{can}^{*}\left(\kappa \right)\right)-{∆\varphi }_{N}\left(\vartheta_{can}^{*}\left(\kappa \right)\right)$ from both sides, the inequality is rewritten as,

(45) $$\psi \left(\theta \right)-∆\psi_{N}\left(\theta \right)-\left(\theta -∆\theta \right)\cdot \vartheta_{can}^{*}\left(\kappa \right)\;\geq \psi \left(\vartheta_{can}\left(\lambda \right)\right)-∆\psi_{N}\left(\vartheta_{can}\left(\lambda \right)\right)-\left(\vartheta_{can}\left(\lambda \right)-∆\vartheta_{can}\left(\lambda \right)\right)\cdot \vartheta_{can}^{*}\left(\kappa \right),$$

where we used (24). From the iso-dual-energy condition in (42), we rigorously obtained the following inequality,

(46) $$\psi \left(\theta \right)-∆\psi_{N}\left(\theta \right)\geq \psi \left(\vartheta_{can}\left(\lambda \right)\right)-∆\psi_{N}\left(\vartheta_{can}\left(\lambda \right)\right).$$

The minimum Massieu-potential principle is derived in the limit of large N,

(47) $$\psi \left(\theta \right)\geq \psi \left(\vartheta_{can}\left(\lambda \right)\right).$$

## 5. Application to a Simple Chaotic Map System

We will reconsider the time evolution of a simple chaotic map system from the information-geometric viewpoint.

**Figure 3:**
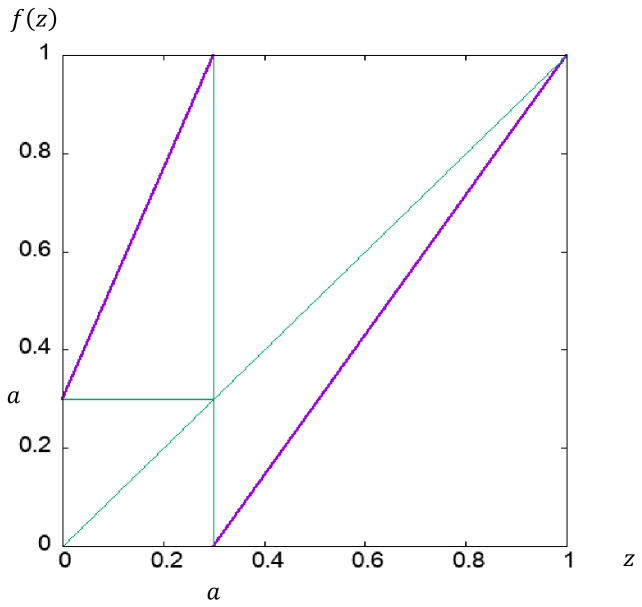
Purple lines: the piecewise linear function $f\left(z\right)$, defined as $\left(1-a\right)\frac{z}{a}+a\;\;\;if\;z\in \left[0,a\right)$ and $\frac{z-a}{1-a}\;if\;z\in \left[a,1\right)$. Green lines: auxiliary lines.

We consider the following chaotic map of the unit interval into itself, satisfying the following equation:

(48) $$z_{t+1\;}=f\left(z_{t\;}\right)=\left\{\begin{array}{ll} \left(1-a\right)\frac{z_{t}}{a}+a & if\;z_{t}\in \left[0,a\right)\\ \frac{z_{t}-a}{1-a} & if\;z_{t}\in \left[a,1\right) \end{array},\right.$$

where 0<a<1 , as is illustrated in Figure 3. The map is a kind of modified Bernoulli map. Similar maps were discussed in the analysis of chaos in dynamical systems [14,15].

The time evolution of a probability density is given as,

(49) $$\rho_{t+1\;}\left(z\right)=L\rho_{t}\left(z\right),$$

where the Frobenius—Perron operator is written as,

(50) $$L\rho \left(z\right)=\left\{\begin{array}{ll} \left(1-a\right)\rho \left(\left(1-a\right)z+a\right) & \;\;\;\;\;\;\;if\;z\in \left[0,a\right)\\ \frac{a}{1-a}\rho \left(\frac{a}{1-a}\left(z-a\right)\right)+\left(1-a\right)\rho \left(\left(1-a\right)z+a\right) & \;\;\;\;\;\;\;if\;z\in \left[a,1\right) \end{array}\right..$$

The invariant density is uniform on each of the two intervals,

(51) $$\rho_{inv}\left(z\right)=\left\{\begin{array}{ll} \frac{1}{1+a} & \;\;\;\;\;\;\;if\;z\in \left[0,a\right)\;\\ \frac{1}{1-a^{2}} & \;\;\;\;\;\;\;if\;z\in \left[a,1\right) \end{array}\right..$$

To introduce $p_{x}$ (x=0,1,…,n) in the chaotic system, we define n+1 intervals as follows,

(52) $$I_{x}=\left\{\begin{array}{ll} \left[0,a\right) & \;\;\;\;\;\;\;if\;x=0\\ \left[a+\frac{1-a}{n}(i-1),a+\frac{1-a}{n}i\right) & \;\;\;\;\;\;\;if\;x=i,\;\;i=1,2,\ldots ,n \end{array}\right.,$$

where the second interval $\left[a,1\right)$ was divided into n equal parts, for simplicity.

We define,

(53) $$p_{t,x}=Prob\left(z_{t}\in I_{x}\right).$$

Then,

(54) $$p_{inv,0}=\frac{a}{1+a},\;\;\;p_{inv,i}=\frac{1}{1+a}\frac{1}{n},\;i=1,2,\ldots ,n.$$

We also define the reference distribution as,

(55) $$\bar{p}=p_{inv}.$$

We define the dual canonical distribution as a uniform distribution on each of the two intervals $\left[0,a\right)$ and $\left[a,1\right)$. The dual canonical distribution evolves along the dual canonical geodesic in Figure 2 and converges to the invariant distribution [13].

**Figure 4:**
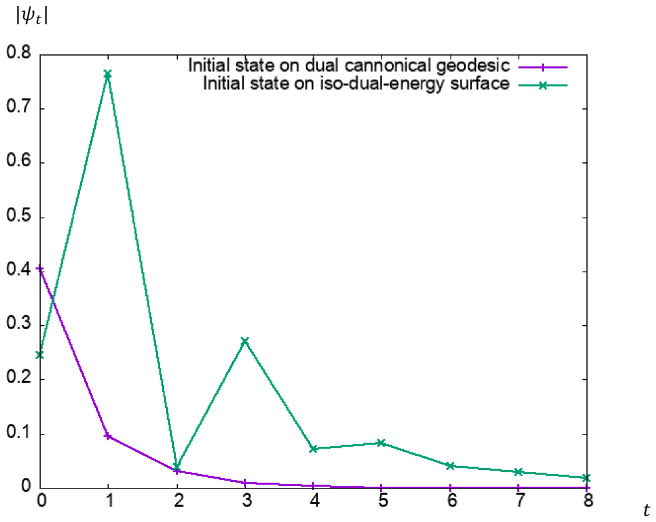
Time evolutions of the absolute value of the Massieu potential at the parameter, a=0.3. The purple line: the initial state on the dual canonical geodesic: the total number of particles, $N=1.3\times 10^{7}$, the initial number of particles in the interval $\left[0,a\right)$, $N_{t=0,0}=4\times 10^{6}$. The green line: the initial state on the iso-dual-energy surface: the total number of particles, $N=1.3\times 10^{7}$, the initial number of particles in the interval $\left[0,a\right)$, $N_{t=0,0}=3\times 10^{6}$, the initial particles are uniformly distributed in the interval $\left[0,a\right)$, the initial number of particles in the interval $\left[a,1\right)$, $\sum_{i=1}^{n}N_{t=0,i}=1.0\times 10^{7}$, the initial particles are distributed exponentially as exp[−4 z] in the interval $\left[a,1\right)$.

When the initial distribution is chosen as the dual canonical distribution, the absolute value of the Massieu potential exponentially converges to zero, which is that of the invariant distribution. As shown by the purple line in Figure 4, the smooth convergence is numerically confirmed. The Massieu potential is written as

(56) $$\psi_{t}=-\log\left(\frac{p_{t,0}}{p_{inv,0}}\right)=-\log\left(\frac{\left(1+a\right)N_{t,0}}{aN}\right),$$

where N is the total number of particles and $N_{t,0}$ is the number of particles in the interval $\left[0,a\right)$ at time t. It is easy to estimate the Massieu potential by just counting the number of particles, $N_{t,0}$.

Next, we consider the time evolution of the following non-uniform initial density,

(57) $$\rho_{t=0}\left(z\right)=\left\{\begin{array}{ll} e^{-\psi_{t=0}}\rho_{inv}\left(z\right) & \;\;\;\;\;\;\;z<a\\ e^{\theta_{t=0}(z)-\psi_{t=0}}\rho_{inv}\left(z\right) & \;\;\;\;\;\;\;z>a \end{array}\right.,$$

where we choose,

(58) $$\theta_{t=0}\left(z\right)=-\beta \left(z-\frac{1+a}{2}\right),$$

so that the initial state lies on the iso-dual-energy surface,

(59) $$\theta_{t=0}\cdot \vartheta_{inv}^{*}=\frac{1}{1-a^{2}}\int_{a}^{1}\theta_{t=0}\left(z\right)dz=0,$$

since $\theta_{t=0}\left(z\right)$ is an anti-symmetric function within the interval $\left[a,1\right)$. We replaced the dot product with the integration.

The initial Massieu potential, $\psi_{t=0}$, is determined by the normalization condition,

(60) $$e^{\psi_{t=0}}=p_{inv,0}+\left(1-p_{inv,0}\right)\int_{a}^{1}e^{\theta_{t=0}\left(z\right)}dz.$$

After integrating the right-hand side, we obtain,

(61) $$\psi_{t=0}=\log\left[1+\frac{1}{1+a}\left\{\frac{2}{\beta \left(1-a\right)}\sinh\left\{\frac{\beta }{2}\left(1-a\right)\right\}-1\right\}\right]=\frac{1}{6}\frac{1}{1+a}{\left\{\frac{\beta }{2}\left(1-a\right)\right\}}^{2}-\ldots .$$

Then,

(62) $$p_{t=0,0}=\frac{1}{1+\frac{2}{a\beta (1-a)}\sinh\left\{\frac{\beta }{2}\left(1-a\right)\right\}}.$$

In the numerical simulation, we prepared a total of N particles at t=0. A total of $p_{t=0,0}N$ particles are uniformly distributed in the interval [0,a) while the remaining particles are distributed exponentially as exp[−βz] in the interval [a,1). In this case, the following behavior can be expected: if the system moves on the iso-dual-energy surface orthogonal to the dual canonical geodesic line, the Massieu potential smoothly decreases to its minimum value of zero.

As shown by the green line in Figure 4, this non-uniform initial distribution causes a large change in the particle number in the interval [0,a) at t=1, resulting in a significant jump in the absolute value of the Massieu potential. This implies that the state has deviated significantly from the iso-dual-energy surface. Although the state subsequently converges to the invariant distribution, its decay profile differs from the one along the dual canonical geodesic, suggesting a different underlying mechanism of decay.

## 6. Conclusions and Remarks

In this paper, we introduce the dual probability distribution of the n-dimensional simplex $S_{n}$. According to Sanov’s Lemma for the multinomial distribution, the dual potential can be interpreted as an exponent of the sampling distribution generated by the reference distribution. The integral representation of this exponent introduces new stochastic variables that follow the dual probability distribution.

The dual probability distribution yields asymptotically dual-symmetric correspondences. The dual potential can be expressed as a cumulant generating function with respect to the dual reference distribution. The parameter vector is obtained by averaging the dual potential using the inverse Legendre transformation. The Massieu potential is equal to the KL divergence between the dual probability distribution and the dual reference distribution.

Information-theoretic principles, such as the maximum-entropy and minimum Massieu-potential principles, can be derived asymptotically from the dual-probability-distribution perspective. For the minimum Massieu potential principle, we introduced a dual canonical distribution explicitly, whose exponent is written using one dual parameter and a dual energy function. The minimum Massieu potential principle is derived asymptotically from the Pythagorean theorem, based on the orthogonality between the dual canonical geodesic and the iso-dual-energy surface.

We expect new insights to play an important role in data analysis, given that dual probability distributions naturally arise in data sampling. Specifically, we intend to apply our argument to the analysis of time series for simple chaotic systems [10], the probability distribution of which evolves according to a gradient flow equation [11,12,13]. In this paper, we present the results of applying our discussion to a simple chaotic map system. We simulate the time evolution for two initial states: One is on the dual canonical geodesic, and the other is on the iso-dual-energy surface. The time evolution of the former initial state is comprehensible because it follows the gradient flow equation. However, the latter is difficult to understand because the dual-energy conservation does not hold.

Finally, this paper provides comments on future research. Our arguments can be easily applied to the maximum work formulation (the dual formulation) using KL divergences scaled by the canonical parameter (the dual-canonical parameter) [16]. The Pythagorean theorem for the scaled divergences gives orthogonality between the canonical-parameter geodesic and the isentropic surface (the dual canonical-parameter geodesic and the iso-Massieu-potential surface). We expect that the information-geometric perspective gives us a clear understanding of the time evolution of chaotic systems in an isentropic process (iso-Massieu potential process).

## Data Availability

No new data were created or analyzed in this study. Data sharing is not applicable to this article.

## Appendix A. Dual Probability Distribution

Suppose N independent data $x_{\tau }$ are generated from the reference distribution, $\bar{p}$, in $S_{n}$. The empirical distribution of the data is given by p,

(A1) $$p_{x}=\frac{1}{N}\sum_{\tau =1}^{N}\delta_{x_{\tau },x}=\frac{N_{x}}{N},$$

we also define ${\bar{p}}_{x}={\bar{N}}_{x}/N.$ Then, the following Lemma is asymptotically valid.

Sanov’s Lemma: the probability of p is asymptotically given by

(A2) $$Prob\{p:\bar{p}\}=\exp\{-ND_{KL}\left[p:\bar{p}\right]\}.$$

Then, the dual potential $\varphi \left(\theta^{*}\right)=D_{KL}\left[p:\bar{p}\right]$ is rewritten as follows,

(A3) $$\varphi \left(\theta^{*}\right)=\sum_{x=0}^{n}\frac{N_{x}}{N}\log\left(\frac{N_{x}}{{\bar{N}}_{x}}\right)=1+\frac{1}{N}\sum_{x=0}^{n}\{\log\left(N_{x}!\right)-\log({\bar{N}}_{x}^{N_{x}})-\frac{1}{2}\log\left(2\pi N\right)\}+{∆\varphi }_{N}\left(\theta^{*}\right),$$

where we define

(A4) $${∆\varphi }_{N}\left(\theta^{*}\right)\equiv -\frac{1}{N}\sum_{x=0}^{n}\left\{\log[N_{x}!]-N_{x}\log N_{x}+N_{x}-\frac{1}{2}\log\left(2\pi N\right)\right\}.$$

From Robbins’ bound, $N!=\sqrt{2\pi N}{\left(\frac{N}{e}\right)}^{N}e^{\lambda_{N}}$, where $\frac{1}{12N+1}<\lambda_{N}<\frac{1}{12N}$,

(A5) $${∆\varphi }_{N}\left(\theta^{*}\right)=-\frac{1}{N}\sum_{x=0}^{n}\left\{\frac{1}{2}\log p_{x}+\lambda_{N_{x}}\right\}=O\left(\frac{1}{N}\right).$$

From the integral representation of the Gamma function,

(A6) $$N_{x}!=\Gamma \left(N_{x}+1\right)=\int_{0}^{\infty }t^{N_{x}}e^{-t}dt=\int_{-\infty }^{\infty }e^{N_{x}y_{x}}e^{y_{x}-e^{y_{x}}}dy_{x},$$

we obtain the following cumulant generating function with respect to $p_{x}$,

(A7) $$\varphi \left(\theta^{*}\right)-{∆\varphi }_{N}\left(\theta^{*}\right)-1+\frac{n+1}{2N}\log\left(2\pi N\right)=\frac{1}{N}\log E_{\tilde{P}\left(y\right)}\left[e^{N\left(p_{0}y_{0}+p\cdot y\right)}\right],$$

where

(A8) $$E_{\tilde{P}\left(y\right)}\left[\cdot \right]\equiv {∭}_{-\infty }^{\infty }\cdot \tilde{P}\left(y\right)dy_{0}\ldots dy_{n},$$

(A9) $$\tilde{P}\left(y\right)\equiv e^{\sum_{x}\{y_{x}+\log(N{\bar{p}}_{x})-e^{y_{x}+\log(N{\bar{p}}_{x})}\}}.$$

It is easy to confirm that $E_{\tilde{P}\left(y\right)}\left[1\right]=1$.

We need to rewrite (A7) to obtain the cumulant generating function with respect to the dual parameters, $\theta_{i}^{*}=p_{i}\;(i=1,\ldots ,n)$. Since, $p_{0}=1-\sum_{i}p_{i}$, $p_{0}y_{0}+p\cdot y=y_{0}+\sum_{i}p_{i}\left(y_{i}-y_{0}\right)\equiv y_{0}+\sum_{i}p_{i}{\tilde{y}}_{i}$. After straightforward calculation,

(A10) $$\varphi \left(\theta^{*}\right)-{∆\varphi }_{N}\left(\theta^{*}\right)-c_{N}=\frac{1}{N}\log E_{\bar{P}\left(y\right)}\left[e^{N\theta^{*}\cdot \tilde{y}}\right],$$

where the dual reference distribution is

(A11) $$\bar{P}\left(y\right)\equiv \frac{N^{N}}{N!}{\bar{p}}_{0}^{N}e^{{Ny}_{0}}\tilde{P}\left(y\right),$$

and the constant $c_{N}$ is defined as,

(A12) $$c_{N}\equiv -\log{\bar{p}}_{0}-\frac{n+1}{2N}\log\left(2\pi N\right)+1-\log N+\frac{1}{N}\log\left(N!\right).$$

It is easy to confirm that $E_{\bar{P}\left(y\right)}\left[1\right]=1$.

From (A11) and (A12), we finally obtain the dual probability distribution,

(A13) $$P\left(y|\theta^{*}\right)\equiv e^{N\left\{\theta^{*}\cdot \tilde{y}-\varphi \left(\theta^{*}\right)+{∆\varphi }_{N}\left(\theta^{*}\right)+c_{N}\right\}}\bar{P}\left(y\right).$$
